# A Close Clinical Workup of a Spontaneous Idiopathic Hepatic Rupture

**DOI:** 10.7759/cureus.61966

**Published:** 2024-06-08

**Authors:** Danis Lester, Ahmad Abdel-Aty, Joshua Hagan

**Affiliations:** 1 Surgery, University of Central Florida College of Medicine, Ocala, USA; 2 Internal Medicine, University of Central Florida College of Medicine, Ocala, USA; 3 Trauma and Acute Care Surgery, HCA Florida Ocala Hospital, Ocala, USA

**Keywords:** hepatic rupture, spontaneous idiopathic hepatic rupture, atraumatic hepatic rupture, idiopathic hepatic rupture, spontaneous hepatic rupture

## Abstract

Atraumatic spontaneous liver rupture is a very rare occurrence. Most case reports and case series focus on patients during pregnancy, conditions associated with malignancy, hepatomegaly/hepatic pathology, benign masses/lesions, or infectious etiologies. This case report presents a unique circumstance where none of the above-mentioned etiologies were evident at the initial presentation or during the clinical workup. The patient presented with some non-specific symptoms of biliary colic without a conclusive diagnosis before the hepatic rupture. Given the high morbidity and mortality associated with spontaneous liver rupture, we believe this case allows for a closer look at the pre-rupture presentation and eventual sequelae not mentioned elsewhere in the literature.

## Introduction

Atraumatic spontaneous hepatic rupture has been associated with many etiologies but the occurrence remains rare [1]. The exact pathophysiology has yet to be precisely identified. Atraumatic spontaneous hepatic rupture is more commonly associated with hemolysis, elevated liver enzymes, low platelet count (HELLP) syndrome; other causes include benign lesions, malignancy, vascular anomalies, infections, connective tissue disease, and possible association with steroids and other medications. Mortality and morbidity are high in this patient population since the rupture occurs most commonly on an outpatient basis. Therefore, a high degree of suspicion is recommended, as spontaneous hepatic rupture is hard to anticipate. Depending on the patient’s hemodynamic status treatment modalities include observation, hepatic artery ligation, packing, drain placement, embolization of hepatic artery, closure of the damaged hepatic tissue, hepatectomy, or hepatic lobectomy [1].

## Case presentation

A 72-year-old female with a past medical history of ulcerative colitis, psoriatic arthritis, rheumatoid arthritis, hypothyroidism, chronic kidney disease (CKD) stage 3, and hypertension presented to the emergency room due to progressively worsening abdominal pain. The patient reported that two days prior to her presentation, she developed right upper quadrant and epigastric pain after eating a fatty meal. The pain resolved after the patient took dicyclomine, bismuth subsalicylate, and calcium carbonate tablets. The following day, after consuming a fatty meal again, she developed a similar pain that was more persistent. Her pain was described as a stabbing pain that was 10/10 in severity. Additionally, she endorsed several weeks of generalized pruritus. She denied fever, chills, nausea, vomiting, diarrhea, chest pain, or shortness of breath. She also denied a history of any similar episodes. The patient’s last flare of ulcerative colitis was two years prior and was not similar to her current symptoms. Her ulcerative colitis was reportedly well controlled on infliximab and mesalamine, and the patient denied prior extraintestinal symptoms.

**Figure 1:**
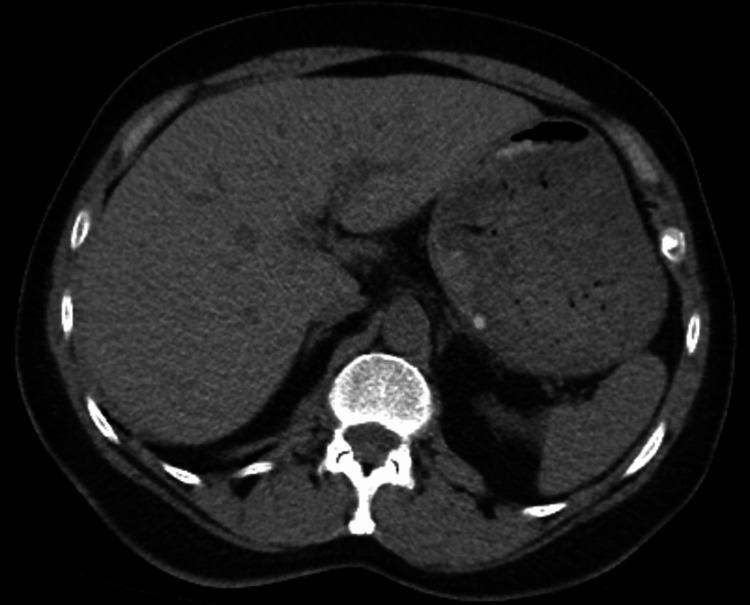
Hospital Day 1: CT abdomen and pelvis without contrast

Upon presentation, the patient was afebrile, with a heart rate of 74 bpm, blood pressure of 148/77 mmHg, and oxygen saturation (SpO_2_) of 94% on room air. The initial exam was notable for tenderness of the epigastrium and right upper quadrant. Her abdomen was soft and non-distended. Murphy’s sign was negative. Initial laboratory testing was remarkable for a WBC count of 13.8 x 10^3^/µL (reference range 4.0 - 10.0 x 10^3^/µL), elevated aspartate aminotransferase (AST) and alanine aminotransferase (ALT) levels, but alkaline phosphatase and total bilirubin were within range. CT abdomen and pelvis without contrast showed subtle peri-portal edema but no hepatic masses or lesions (Figure 1).

**Figure 2:**
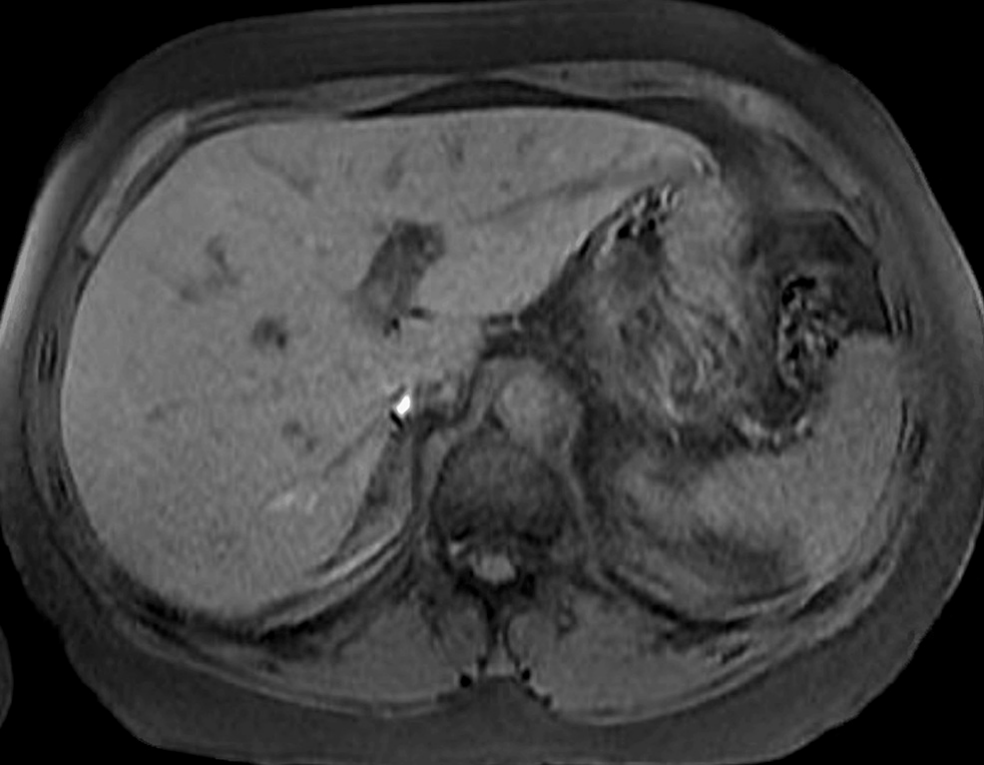
Hospital day 2: MRI MRCP T1 phase MRCP: magnetic resonance cholangiopancreatography

The right upper quadrant ultrasound was unremarkable and showed no evidence of gallbladder stones or sludge, wall thickening, or peri-cholecystic fluid. Magnetic resonance cholangiopancreatography (MRCP) showed no intrahepatic or extrahepatic biliary ductal dilatation (Figure 2).

A hepatobiliary iminodiacetic acid (HIDA) scan demonstrated the radiotracer remaining in the liver on 24-hour delayed images after the administration of 10.1 mCi of Technetium-99.

**Figure 3:**
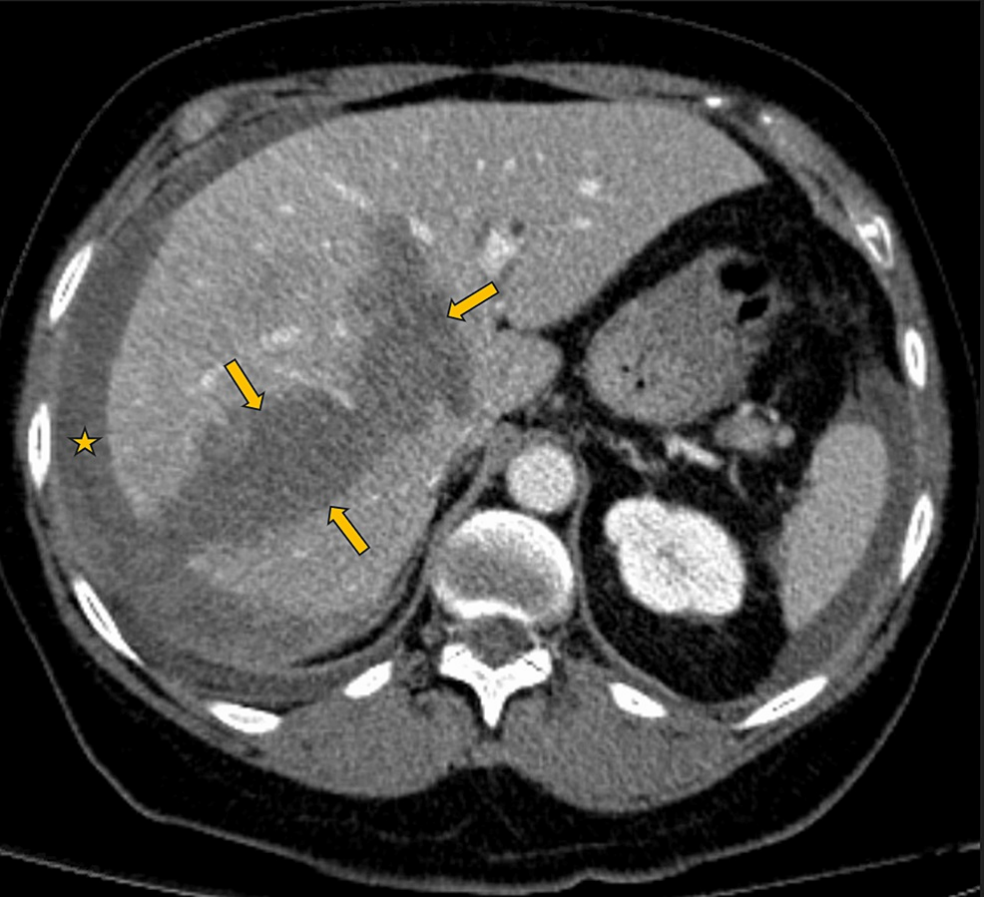
Hospital day 4: CT angiogram of abdomen and pelvis - venous phase Arrows - identify the edges of the hepatic laceration/hematoma; Star - extrahepatic hematoma

The patient’s pain initially improved, and her diet was advanced. On hospital day four, the patient reported an acute onset of worsened right upper quadrant pain after experiencing a "popping-like sensation” in her abdomen. Blood pressure declined to 60s/40s mmHg. On exam, the patient developed diffuse tenderness that was most severe in the right upper quadrant, rebound tenderness, and guarding in the right upper quadrant. Emergent CT and CT angiography of the abdomen and pelvis demonstrated disruption of the liver capsule, a large hypodense area involving the right lobe of the liver and portions of the left lobe of the liver, and a free fluid in the right lower quadrant of the abdomen consistent with acute hepatic rupture with acute hemoperitoneum (Figure 3).

**Figure 4:**
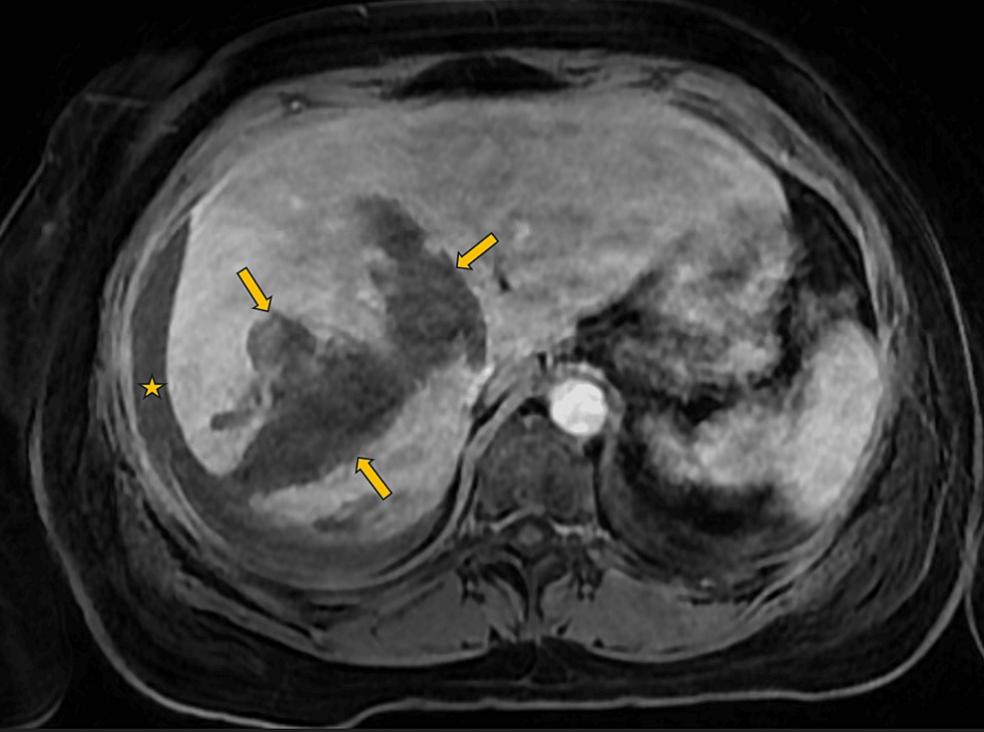
Hospital day 7: MRI abdomen and pelvis T1 phase Arrows - identify the edges of the hepatic laceration/hematoma; Star - extrahepatic hematoma

The patient had no reported recent history of trauma to the abdomen. Based on laboratory values, the patient was transfused two units of fresh frozen plasma, one unit of platelets, and two units of packed red blood cells. Vitals stabilized and there was no evidence of further bleeding. Subsequent MRI with and without intravenous contrast demonstrated unchanged right hepatic lobe laceration and perihepatic blood products (Figure 4).

The patient’s transaminases, alkaline phosphatase, and bilirubin increased and peaked on day four. The patient developed jaundice and scleral icterus. The laboratory values of the patient on admission, peak values, and values on discharge are presented in Table 1.

**Table 1:** Liver function test values during the hospital course aspartate aminotransferase (AST); alanine aminotransferase (ALT)

Laboratory test	On admission	Peak value during hospitalization	On discharge	Reference value
AST	124 IU/L	>1500 IU/L	114 IU/L	12-37 IU/L
ALT	85 Unit/L	1029 Unit/L	131 Unit/L	4-35 Unit/L
Alkaline phosphatase	81 IU/L	221 IU/L	232 IU/L	38-126 IU/L
Total bilirubin	0.8 mg/dL	8.1 mg/dL	1.5 mg/dL	0.2-1.5 mg/dL

Total bilirubin returned to within normal limits by the time of discharge.

The patient was readmitted one month later for shortness of breath due to a large-volume, right-sided pleural effusion. The patient underwent a diagnostic and therapeutic thoracentesis with the removal of one liter of brown-colored fluid likely representing a sympathetic effusion. As cytology and culture were both negative.

One month post-re-admission, the patient followed up with her gastroenterologist and was doing overall well. No further imaging studies have been done to date.

## Discussion

In the adult population, many atraumatic etiologies have been associated with spontaneous liver rupture and spontaneous hepatic hemorrhage. The broad categories of these etiologies are pregnancy-associated (HELLP, acute fatty liver), malignancy (HCC, angiosarcoma, hemangioendothelioma, metastatic disease), benign (adenoma, focal nodular hyperplasia, hemangioma, cystadenoma, angiomyolipoma), vascular anomalies (peliosis hepatis), connective tissue disease (amyloid, SLE, polyarteritis nodosa), infectious (malaria), and hyper-eosinophilic syndrome. Other conditions, such as extensive vomiting and warfarin therapy, have been postulated to have an association [2,3]. In this instance, malignancy, benign, and vascular anomalies are unlikely given no evidence of suspicious lesions on MRI and CT prior to rupture. Despite the patient not having laboratory workup for connective tissue disorders or biopsies, there was no evidence of connective tissue disorder from past medical history or physical examination. The hyper-eosinophilic syndrome may be ruled out as an etiology since the patient's eosinophils were not elevated during hospitalization.

High morbidity and mortality are associated with spontaneous liver rupture. It is usually seen in pregnant women with HELLP syndrome [3]. According to a report by Henny et al., 80% of cases of hepatic rupture in pregnancy occur in multiparous patients with an average age >30 years [4]. The right lobe is more like to be affected than any other lobe of the liver. Parenchymal and subcapsular bleeding and hematoma formation precede hepatic rupture [1]. Due to liver failure from hepatic necrosis in HELLP syndrome, 75% of patients with spontaneous hepatic rupture and hemorrhage end up dying during their index of admission, leading to the highest rate of mortality of all patients with spontaneous hepatic ruptures [2].

No specific pathophysiological mechanisms leading to atraumatic spontaneous hepatic rupture have been identified to date, but various hypotheses have been postulated regarding certain pathologic causes. It is hypothesized that in patients with HELLP syndrome, vasospasm during pregnancy in combination with endothelial vascular damage may lead to the formation of microthrombi that lead to hepatic rupture [5]. In amyloidosis, the formation of thromboses may lead to hepatomegaly, fragile hepatic parenchyma, and unstable vascularity [6]. Hepatocellular carcinoma (HCC) is the most common primary tumor of the liver, and the incidence of spontaneous hepatic rupture is about 10% [7]. A more recent retrospective study performed by Obeidat and Wong in 2022 showed that the incidence of spontaneous hepatic rupture in HCC patients was 3.9%, and the mean tumor size associated with rupture was 8.0 cm [8]. In HCC and other malignant liver lesions, spontaneous liver rupture may be caused by the expanding tumor growth, which ultimately leads to the destruction and fragility of surrounding healthy parenchyma deeming it unstable, or rapidly growing tumors with central necrosis may develop an expanding central hematoma [2,9].

A study conducted by Battula et al. revealed that over 90% of patients with spontaneous hepatic rupture present with non-specific symptoms, of which vague epigastric and right upper quadrant pain, malaise, and vomiting were most common. Hematemesis, chest pain, fever, and jaundice occurred in <10% of patients, whereas 12% of patients had signs of shock on admission [10]. Due to a low index of suspicion and rarity of occurrence, the diagnosis is often delayed [7]. A computed tomographic (CT) scan remains the gold standard diagnostic procedure [2].

Treatment options for spontaneous hepatic ruptures include nonoperative management (e.g. observation), minimally invasive interventions (e.g. hepatic artery ligation, percutaneous drain placement, embolization of hepatic artery), or surgical intervention (e.g. packing, closure of the damaged hepatic tissue, hepatectomy, hepatic lobectomy) [1]. In cases of hepatic rupture caused by a lesion, commonly, after achieving hemostasis and hemodynamic stability, formal staging and assessment of liver function are done prior to hepatectomy of the liver tumors, and these patients primarily have HCC [2].

## Conclusions

The broad picture reveals that pathogenesis leading to compromised integrity of the parenchyma predisposes to increased spontaneous liver rupture risk. Spontaneous hepatic rupture is associated with high mortality and morbidity; due to its rarity, diagnosis can be delayed. The mainstay of treatment of spontaneous hepatic rupture/hemorrhage is to achieve hemodynamic stability and hemostasis. This case report identifies a patient with biliary colic signs and symptoms leading to hepatic rupture. Our case is unique in that it also presents a patient without a known etiology that may cause the hepatic rupture and signs of only biliary colic (i.e. right upper quadrant pain after meals) thus contributing to existing literature. The addition of this valuable case report can serve to further add to this rare pathology, improvement in management, and possible focus on diagnostic workup. Given that our patient does not fit the criteria of the HELLP syndrome patient, it is of high scientific interest to understand the pathology involved, given it may have commonalities with other etiologies, some being oncologic in nature.

This is the first case report to describe idiopathic hepatic rupture with no evidence of the above-mentioned etiologies in a geriatric patient. This leads to the question of whether future diagnostic imaging protocols should be considered in geriatric populations with non-specific symptoms of biliary colic without a conclusive diagnosis. Another potential investigation of whether conservative management should be considered more often in patients who are responsive to resuscitative measures, as illustrated in this case.
